# Editorial Department

**Published:** 1886-01

**Authors:** 


### EDITORIAL DEPARTMENT.

Dr. Billings in Paris.—The subject which of all others absorbs most the attention of the public at the present moment is hydrophobia, and whether Pasteur's assertion that he has discovered a positive cure for the same by the inoculation of his prepared virus will be verified or not is the question most hotly discussed.

For some time past the illustrious Parisian, whose discoveries in bacteriology have spread his fame over the world, has devoted his entire attention to the study of rabies, its prevention and cure. At the Academy of Medicine in Paris he declared recently that his efforts were crowned with complete success. By inoculating rabbits from the spinal cord of an infected animal and cultivating through successive generations he has obtained cultivations of successively increasing virulence and shortened the period of incubation; and by keeping the spinal cords of these rabbits in perfectly dry tubes for varying periods he can obtain a virus attenuated as desired.

Our readers are acquainted with the circumstances which led to the sending of a number of children to Paris to be placed under the immediate care of the great *savant*, in whose laboratory the question will soon be put to a practical test. They will be pleased to learn that Dr. Billings has been selected to accompany the cases so timely sent from this country to assist M. Pasteur in the experiments and to note in detail the *modus operandi* thereof.

The mission of Dr. Billings is an important one, not only as regards the benefits to be derived by those immediately concerned and ultimately by suffering humanity at large, but also on account of its scientific interest to our profession, and that it will be, if successful, a demonstration of the value of experimental pathology. It will also probably result in the establishment in this country of an institution in which pathological research can be carried on as effectually as in Europe, and where mad-dog bitten unfortunates can be treated without being subjected to the delay and expense of an ocean voyage. In such event Dr. Billings will have obtained the necessary experience to organize and direct it. C.

---

The Pyramid Tract.—In presenting an article on the comparative anatomy of the Pyramid Tract, from the pen of a leading American authority on the brain, we desire to direct attention to the fact, that it is intended to serve as a forerunner of a monograph on the brain of the horse, which is now being prepared by a well known veterinarian, on the basis of special anatomical and pathological investigations. There is some reason to believe that our veterinary text-books, in describing the symptoms of paralysis from brain disease in horses, follow certain conventional notions derived from text-books on human medicine, rather than actual observation on horses. It is true that, practically speaking, the elucidation of the subtler pathology of equine paralysis can scarcely hope to become as important a subject to the veterinarian as paralysis of the human subject is to the physician, since a paralyzed horse is, as a rule, permanently crippled and consequently doomed. Yet there are certain questions of diagnosis which may have an important medico-legal, if no other bearing. The determination of the nature of a poison, producing paralysis for example, may depend, during the life of the animal, on the type of the paralysis, whether this be spinal or cerebral. Now, what is the spinal as distinguished from the cerebral type of paralysis in the horse? Do any of our text-books satisfactorily answer that question? It is true that paralysis of one side (hemiphlegia) is represented in such works as the typical result of disease of one hemisphere, and paralysis of both sides is the usual result of disease lower down. But the determination by Professor Spitzka of the fact that the hoofed animals have no true pyramid decussation, is calculated to throw some doubt on the usually accepted criteria. In order to set these doubts at rest, the number of cases in which there is paralysis either of cerebral or spinal origin, cannot be too large. We therefore would ask our subscribers and other friends to carefully note cases of this character, to obtain autopsies wherever possible, and if they lack the time or inclination to make a minute analysis of the nervous apparatus, we shall be pleased to receive the specimens, and submit them to the investigator referred to. Full acknowledgment for histories furnished, and specimens sent, will be made. The latter should be removed from the animal shortly after death. The animal should be killed by bleeding, not by shooting or poisoning. The work of removing the brain and cord is difficult, but faithful compliance with such directions as Mr. McFaydean's work gives will enable the dissector to obtain them intact.

A large vessel, holding at least a gallon, should be filled with a solution of bichromate of potassa, of thirty grains to the ounce of water (about an ounce of the salt to each pint of fluid), or with the solution known as Muller's fluid. A layer of antiseptic cotton should be placed at the bottom of this vessel, and then the brain, or brain and cord, submerged in it. The membranes which surround the brain should be nicked with a scissors wherever accessible, particularly between the cerebellum and oblongata.

The spinal cord should be cut, under the fluid, crosswise into as many pieces as there are pairs of spinal nerves, each segment corresponding to one such pair.

After being preserved for one week in a cool place, undisturbed, the tin case is to be closed, soldered tightly, and sent by express to the Zoölogical Department of Central Park, New York city. If the distance be great, the jar is to be filled with soaked cotton, to prevent excessive shaking. C.

---

Barry's Clinical Thermometer.—We desire to call our readers' attention to the advertising pages where the above instrument is described.

The importance of accurately noting bodily temperature in fevers and inflammations which is so well established in human medicine is equally so when these conditions exist in animals. Every veterinarian then should possess a good thermometer, and we recommend this one as nearest approaching perfection.

---

The Putnam Nail Co., Boston, Mass.—It is generally by inattention to small matters in the care of horses that many valuable animals are lost. A nail is apparently an insignificant affair, yet a defective one will lame your horse. Great danger of lameness is often incurred in shoeing by using nails which split in driving. The Putnam nail is the only one in which this does not take place, and the most experienced horsemen concede it the highest place among nails.

---

Testimonial.—The Faculty of Students of the American Veterinary College, in acknowledgment to Dr. Billings, previous to his departure for Europe, of their appreciation of the interest always displayed by him whilst connected with their institution, presented him with a substantial testimonial of their kind wishes. C.

